# TRAJECTORIES OF FATIGUE AND RELATED OUTCOMES FOLLOWING MILD ACQUIRED BRAIN INJURY: A MULTIVARIATE LATENT CLASS GROWTH ANALYSIS

**DOI:** 10.2340/jrm.v56.32394

**Published:** 2024-03-20

**Authors:** Tom SMEJKA, Daan VERBERNE, Jan SCHEPERS, Claire WOLFS, Vera SCHEPERS, Rudolf PONDS, Caroline VAN HEUGTEN

**Affiliations:** 1 Department of Neuropsychology and Psychopharmacology, Faculty of Psychology and Neuroscience, Maastricht University, Maastricht; 2 Limburg Brain Injury Centre; 3 Department of Neurorehabilitation, Sint Maartenskliniek, Nijmegen; 4 Department of Methodology and Statistics, Faculty of Psychology and Neuroscience, Maastricht University, Maastricht; 5 School for Mental Health and Neuroscience, Faculty of Health, Medicine and Life Sciences, Maastricht University, Maastricht; 6 Department of Rehabilitation, Physical Therapy Science and Sports, University Medical Center Utrecht, Utrecht; 7 Department of Medical Psychology, Amsterdam University Medical Center, Location VU, Amsterdam, the Netherlands

**Keywords:** fatigue, latent class growth analysis, stroke, traumatic brain injury

## Abstract

**Objective:**

Fatigue is a common symptom following acquired brain injury although the severity and course differs for many individuals. This longitudinal study aimed to identify latent trajectory classes of fatigue and associated outcomes following mild brain injury.

**Methods:**

204 adults with mild traumatic brain injury (159; 78%) or minor stroke (45; 22%) were assessed 4 times over 1 year. Subjective measures of fatigue, anxiety, depression, cognitive complaints and societal participation were collected. Multivariate Latent Class Growth Analysis identified classes of participants with similar longitudinal patterns. Demographic and injury characteristics were used to predict class membership.

**Results:**

Analysis revealed four classes. Class 1 (53%) had mild, decreasing fatigue with no other problems. Class 2 (29%) experienced high persistent fatigue, moderate cognitive complaints and societal participation problems. Class 3 (11%) had high persistent fatigue with anxiety, depression, cognitive complaints and participation problems. Class 4 (7%) experienced decreasing fatigue with anxiety and depression but no cognitive or participation problems. Women and older individuals were more likely to be in class 2.

**Conclusion:**

Half the participants had a favourable outcome while the remaining classes were characterised by persistent fatigue with cognitive complaints (class 2), decreasing fatigue with mood problems (class 4) or fatigue with both cognitive and mood problems (class 3). Fatigue treatment should target combinations of problems in such individual trajectories after mild brain injury.

LAY ABSTRACT

Many people experience ongoing fatigue after a mild brain injury. We followed 204 adults with mild traumatic brain injury or minor strokes to investigate whether fatigue and associated outcomes (anxiety, depression, cognitive complaints and societal participation) follow similar patterns and whether factors like age, gender, education and type of injury influence who follows which patterns. We assessed the participants’ symptoms at four points: 6 weeks, 3, 6 and 12 months after their injury. We used statistical analyses to group people with similar patterns of symptoms and outcomes and found different groups. Half of the people had mild decreasing fatigue and few additional problems, while the others had high to moderate fatigue with mood and/or cognitive problems. Women and older individuals were more likely to experience high fatigue alongside cognitive and participation problems. Treatment of fatigue should be combined with other treatments, e.g. mood improvement, targeted to an individual’s profile.

Fatigue is not only one of the most prevalent symptoms following mild traumatic brain injury (mTBI) and minor stroke (1, 2), but is subjectively rated as the most severe of the remaining symptoms up to 3 years post injury (3). While a decline in fatigue is seen at the mean level over 12 months, some individuals have been shown to develop fatigue between 6 and 12 months, potentially due to social changes such as returning to work (4). Given the individual differences in fatigue rates over 12 months, it is important to understand how fatigue trajectories differ between individuals with mild acquired brain injury (ABI).

A study by Rakers et al. (5) assessed 456 mTBI patients on fatigue, depression, anxiety, posttraumatic stress and coping style at three time points (2 weeks, 3 months and 6 months post injury). Multivariate Latent Class Growth Analysis (MLCGA) was used to identify classes of individuals that follow similar patterns across different variables over time. In their analysis four classes were identified. The first class (30%) experienced complete recovery within 6 months in combination with an active coping style. The second class (25%) experienced a decline in fatigue and negative mood with a decreasing use of passive coping over time. Classes 3 (27%) and 4 (18%) both showed persistent fatigue, with class 3 showing a trend toward more passive coping over time and with class 4 experiencing both high negative mood and high increasing use of passive coping. Furthermore, patients who had a lower fatigue recovery (classes 3 and 4) tended to be women and those in class 4 were also more likely to have a lower education level.

This study (5) gives valuable insight into the variability of fatigue and other post-injury symptoms over time. However, given the potential social changes between 6 and 12 months (e.g. return to work) (4), it would be valuable to explore the symptom trajectories up to 12 months. Furthermore, as fatigue affects both mTBI and minor stroke groups, and they are often treated in the same clinical (rehabilitation) setting, including both groups in analysis could reveal whether there are trajectories unique to one injury type and subsequently whether they should be treated differently.

Depression and anxiety (included in Rakers et al.’s MLCGA model) (5) have been shown to correlate with fatigue in previous studies (1, 4); however, other post-injury symptoms associated with fatigue should be considered when investigating recovery trajectories. Subjective cognitive difficulties have been shown to predict fatigue after TBI (6) while qualitative research has shown that engaging in meaningful social activities helped participants overcome their fatigue (7). While returning to work post injury may increase fatigue (4), some social activities might actually be beneficial in encouraging individuals to engage despite fatigue.

We therefore applied MLCGA to a mixed cohort of mTBI and minor stroke patients over the course of 12 months in relation to fatigue and other related factors after brain injury. The present study had the following research questions:

1. What are the common trajectories of fatigue and associated consequences (anxiety, depression, cognitive complaints and societal participation) following mild brain injury within a mixed cohort of mTBI and minor stroke patients over the course of 12 months?
2. To what extent do the demographic and injury characteristics age, sex, education level and brain injury type predict class membership to specific trajectories?

## METHODS

### Design

Data used in the present study were collected as part of a larger dataset investigating emotional and cognitive recovery following ABI (8). This study was a multicentre, prospective, longitudinal observational cohort study following participants with mild to moderate TBI, stroke or other forms of ABI over the course of 12 months. For analysis in the present study, only participants with mTBI or minor stroke were included. This study was granted approval by the medical ethics committee of Maastricht University Medical Center (MUMC+; 16-4- 181).

### Participants

Recruitment of participants was conducted between April 2017 and October 2018 at 5 hospitals in the south of the Netherlands. Inclusion criteria were:

- Adults (> 18 years of age).
- Mild to moderate ABI within the last 6 weeks as diagnosed by a clinician.
- Discharged home after visiting the emergency department (ED) or hospital admission.

Exclusion criteria were:

- Inability to give informed consent.
- Insufficient capability to follow up for 1 year due to health problems.
- Insufficient level of the Dutch language to complete the questionnaires.

Exclusion criteria were judged by the participant’s clinician.

For the analyses in this paper only mTBI and minor stroke were considered, for which severity was assessed at admission by the Glasgow Coma Scale (GCS) and the National Institutes of Health Stroke Scale (NIHSS) respectively. mTBI was classified by the clinical criteria of the World Health Organization Collaborating Centre Task Force. This includes a GCS of 13-14 or a GCS of 15 and one of the following symptoms: confusion or disorientation, post-traumatic amnesia (< 24 h), loss of consciousness (< 30 min) or other transient neurological symptoms such as focal signs, seizures or intracranial lesions not requiring surgery (9). Minor stroke was defined according to a NIHSS score of 1-4 (10).

### Procedure

Participants were informed of the study at the ED or neurology outpatient clinic by a physician. Those interested were given an information leaflet and their contact details were forwarded to the researcher. Participants were then contacted by phone and subsequently the written study information and consent form were sent to the participants’ homes including the first set of questionnaires.

At each time point participants were sent a set of questionnaires to complete (online or via mail, based on participant preference). The first assessment (T0) took place within the first 6 weeks following brain injury. Subsequent follow-up assessments took place online or via mail at 3 (T1), 6 (T2) and 12 months (T3) post injury. If the signed consent form and first questionnaire were not returned within the 6-week deadline, follow-up assessments were not sent.

### Measures

*Demographic and medical information*. The same measures were collected at each time point with the exception of demographic information and injury characteristics, which were collected only at T0. This information included: age, sex, educational level (low [primary school education], middle [secondary school education] or high [university/vocational training]), type of brain injury and time since injury. Information concerning the cause of TBI and type of stroke as well as severity were collected from medical files.

*Fatigue*. Fatigue was assessed using the Fatigue Severity Scale (FSS) (11). This scale consists of 9 items rated by agreement with the statements concerning fatigue severity on a scale of 1 to 7. These scores are then averaged over the 9 items. Higher scores indicate higher fatigue severity (range 1–7). A score of ≥ 4 is considered clinically significant fatigue (11). Later psychometric research on the FSS found that better validity of the scale is found and changes are more likely to be seen in a stroke population when the first two items are removed (12), so in the current analysis the average score of items 3–9 was used.

*Anxiety and depression*. The Hospital Anxiety and Depression Scale (HADS) consists of 14 items where higher scores indicate more severe symptoms of either depression (HADS-D) or anxiety (HADS-A) (range 0–21). A score of ≥ 8 per subscale indicates clinically significant depression or anxiety symptoms (13). Raw scores for each scale were used in analysis.

*Cognitive problems*. The Checklist for Cognitive and Emotional Consequences of Stroke (CLCE-24) consists of 24 items and covers 2 subscales assessing cognitive and emotional complaints separately. The present study analysed the cognitive complaints only, which are assessed by 13 questions. These are scored by absence or presence of symptoms (0–1). Where participants reported having doubts about the presence of a problem, the problem was scored as present. Higher scores are associated with more cognitive problems in daily life (14). Raw scores (range 0–13) were used in analysis. The questionnaire was adapted to the current study population by replacing “stroke” with “brain injury” where necessary.

*Societal participation*. Participation in society was assessed by the Utrecht Scale for Evaluation and Rehabilitation-Participation (USER-P) (15). This questionnaire comprises 31 items over 3 scales examining: (1) the frequency of participation in social activities, both in hours per week and number of times in a month, (2) whether participation is restricted by the participant’s current condition rated by 11 items on a 4-point scale (not possible–without difficulty) and (3) the participant’s satisfaction with daily life participation rated by 10 items on a 5-point scale (very dissatisfied–very satisfied). Each scale’s score is converted into an outcome score from 0–100 with higher scores indicating more participation (more often, fewer restrictions and higher satisfaction). The scores for restrictions and satisfaction were used in analysis.

While there are no clinical cut-offs on this scale, data in the present study were comparted to mean scores for stroke and ABI groups in previous literature. Mean scores for these groups show restrictions to be between 78 and 81 and satisfaction between 68 and 72 (16, 17).

### Analysis

Data were analysed using Mplus version 7.3 (https://www.statmodel.com/) (18), SPSS version 27 (IBM Corp, Armonk, NY, USA) and R version 4.2.2 (R Foundation for Statistical Computing, Vienna, Austria). MLCGA was performed on the FSS, HADS-A, HADS-D, CLCE-24, USER-P restrictions and USER-P satisfaction, giving a total of 6 variables, which were modelled together. The MLCGA model analyses multivariate change over time to identify latent classes of patients with similar multivariate patterns of recovery. Missing data are handled using full information maximum likelihood, estimating model parameters based on all available data. The percentage of missing data per variable per time point can be found in Table SI.

Five MLCGA models (with 1–5 classes) were estimated. Subsequently, the model with the lowest Bayesian Information Criterion (BIC) and best interpretability of the 5 models was chosen. For the chosen model, each class represents a group of patients with a shared recovery trajectory across the 6 dependent variables. Second-order polynomial curves were estimated to capture these recovery trajectories. Three significance values are given for each curve, the intercept (difference from the other classes at baseline), linear (difference between T0 and T3) and quadratic effect (difference between each time point).

Furthermore, age, sex, education level and brain injury type were included in the MLCGA model as concomitant variables for predicting class membership (where the latent group indicators are assumed multinomial) (19). Where applicable, analyses were performed with a Bonferroni–Holm correction for multiple comparisons.

## RESULTS

### Participants

A total of 316 participants were recruited for the study. Subsequently, 91 were removed for not meeting the criteria for mild ABI, a further 18 participants chose to actively drop out of the study with no reason given and 3 passed away. A final 204 participants were included in the analysis. Table I gives the demographic distributions of the final cohort.

**Table I:** Participant demographics and clinical characteristics

Characteristics	*n*	Participants (*n* = 204) mean (SD), (range) or *N* (%)
Age (years)		57 (17.8), (19–86)
Sex (male)		114 (56%)
Time since injury at T0 (days)		23.8 (11.1), (7–48)
Injury type mTBI Minor stroke		159 (78%) 45 (22%)
Education level		
Low		52 (26%)
Middle		96 (47%)
High		56 (27%)
Fatigue (FSS-7)		
T0		4.3 (1.6), (*N* = 201)
T1		3.8 (1.7), (*N* = 188)
T2		3.7 (1.7), (*N* = 181)
T3		3.5 (1.7), (*N* = 171)
Mild traumatic brain injury, *n*	159	
Cause of injury, *n*	157	
Fall		77 (49%)
Traffic		59 (37%)
Violence		4 (3%)
Sports		9 (6%)
Hit by object		6 (4%)
Other		2 (1%)
Severity (GCS)	159	14.9 (0.4) (14–15)
Loss of consciousness (yes)	152	99 (62%)
Duration in minutes	84	4.8 (6.1), (0.03–30)
PTA (yes)	159	89 (56%)
Duration in hours	82	2 (4.3), (0.02–24)
Other transient neurological symptoms (yes)	159	73 (46%)
Minor stroke	45	
Severity (NIHSS)	45	2 (1), (1–4)
Type of stroke (ischemic)	45	43 (96%)
Hemisphere	45	
Left		17 (38%)
Right		17 (38%)
Other		11 (24%)
Location	44	
Anterior		1 (2%)
Medial		31 (71%)
Posterior		3 (7%)
Vertebrobasilar		9 (20%)
Intravenous thrombolysis (yes)	45	8 (17%)

GCS: Glasgow Coma Scale; NIHSS: National Institutes of Health Stroke Scale; PTA: post-traumatic amnesia. Note: The fatigue scores show the number of participants decreasing over time due to missing data and not dropping out of the study.

### Fatigue

Table I shows that while the mean fatigue score for the whole cohort shows a general decrease over time, there is large individual variability given the high standard deviations. A boxplot displaying the fatigue variability over the 4 time points is shown in Fig. S1 and a comparison of the means and standard deviations on the FSS-7 and FSS-9 can be found in Table SII.

### Classes

**Fig. 1:**
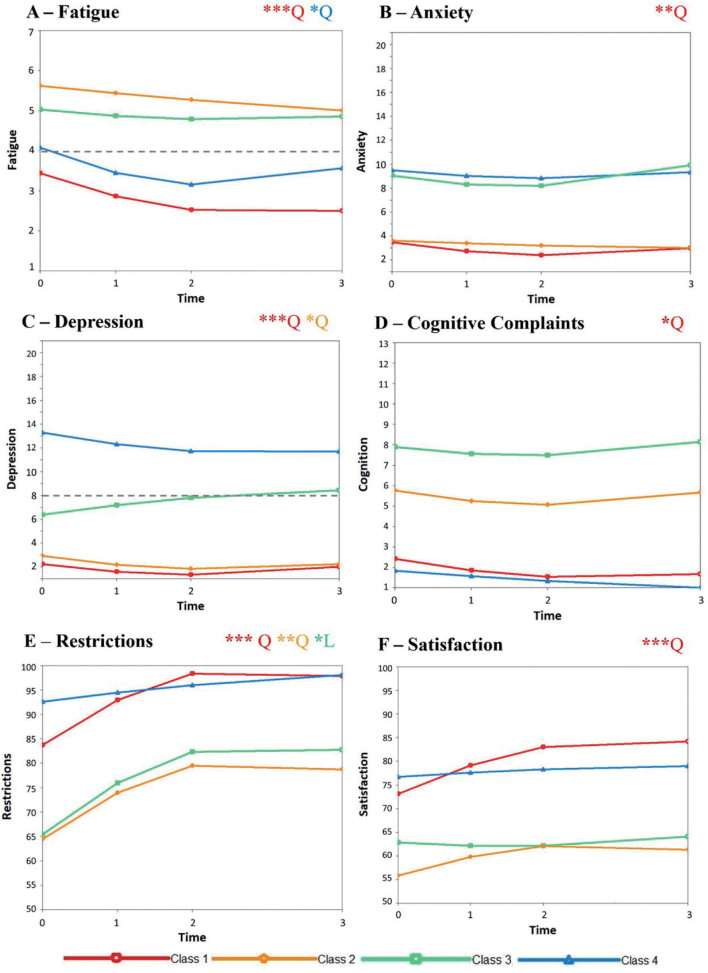
Class trajectories over time per variable. *p < 0.05; **p < 0.01; ***p < 0.001. Q: quadratic; L: linear. Stars are colour coded to represent significance per class. The dashed grey lines in A, B and C represent clinical cut-offs for significant symptoms. Class 1 shows a significant change over time (i.e. linear and quadratic effect) on all variables (A–F). Class 2 showed a significant change over time in depression and restrictions (C & E) and was approaching significance on satisfaction (F) (*p* = 0.053); class 3 had a significant change over time for restrictions (E); class 4 showed a significant change over time for fatigue (A). For restrictions, higher scores indicate fewer restrictions.

**Fig. 2:**
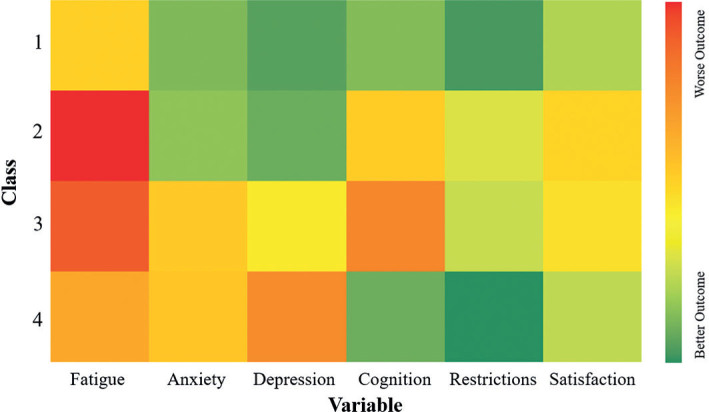
Mean symptom scores by class. Scores for each variable were scaled to fit a range of 0–100. The colours represent the mean score over the 4 time points. Proportions of the sample are divided into classes as follows: class 1 = 53%, class 2 = 29%, class 3 = 11% and class 4 = 7%.

Of the 5 MLCGA models, the model with 4 classes was selected as the best fit for the data as it had the lowest BIC and was interpretable. Fig. 1 shows the class trajectories per variable. The intercept was significant for every variable (*p* < 0.001), which means that at baseline all classes differed significantly from one another on every variable. The significance of the linear and quadratic effect varied for each class and variable. Significant values are described in Fig. 1 and all curve *p*-values can be found in Table SIII. The average score for each variable across all time points per class is shown in Fig. 2.

*Class 1: Mild, decreasing fatigue with no other problems*. This group represented 53% of the sample and showed a good recovery overall. Their initial fatigue was below the cut-off for clinically significant fatigue and also reduced over the 12 months. This group did not meet the cut off for clinically significant anxiety or depression and experienced fewer cognitive complaints than classes 2 and 3. Their initial restrictions and satisfaction scores were both higher than average for this population and continued to improve by 12 months.

*Class 2: High persistent fatigue with primarily cognitive problems*. This group contained 29% of the cohort and showed persistent clinically significant fatigue. This was combined with no mood problems but persistent cognitive complaints. Furthermore, this was associated with the lowest initial restrictions score, although this did improve to an average score for this population by 12 months. Satisfaction in social activities also started and remained lowest of the classes, persistently below the average for an ABI population.

*Class 3: High persistent fatigue with both mood and cognitive problems*. This group was made up of 11% of the participants and experienced clinically relevant, persistent fatigue. Anxiety was persistently mild while depression was consistently borderline clinically relevant. This was combined with the highest persistent cognitive complaints. This group also experienced a low initial restrictions score, although this did improve to an average level by 12 months. Their satisfaction, however, remained persistently below average.

*Class 4: Initially improving moderate fatigue and primarily mood problems*. This group contained 7% of the cohort and showed initial clinically significant fatigue that improved over the first 6 months. This was followed by a mild increase to borderline clinically relevant at 12 months. They also experienced mild persistent anxiety and moderate persistent depression. However, this group had the fewest cognitive complaints across all time points. This was in combination with stable, above-average restrictions and satisfaction scores across the 12 months.

### Predictors

For age it was found that, as compared with younger people, older people were more likely to be in class 2 than class 3: Δ Ln(odds) = 0.041, *p* = 0.036, or class 1: Δ Ln(odds) = 0.03, *p* = 0.042. Sex also predicted class membership, with females, as compared with males, being more likely to be in class 2 than any other class: 2 vs 1: Δ Ln(odds) = 1.456, *p* = 0.004, 2 vs 3: Δ Ln(odds) = 2.144, *p* = 0.005, 2 vs 4: Δ Ln(odds) = 1.574, *p* = 0.011. Education level did not significantly predict membership of any of the classes. However, when comparing high and low education levels, a trend approaching significance revealed that, as compared with higher educated people, lower educated people were more likely to be in class 3 than class 1 (*p* = 0.064). Finally, for brain injury type, there was a higher likelihood for people with mTBI, as compared with minor stroke patients, to be in class 1 than in class 4: Δ Ln(odds) = 1.428, *p* = 0.026. Probabilities for class membership based on demographic characteristics are displayed in Fig. S2.

## DISCUSSION

The current study revealed 4 distinct recovery trajectories of fatigue and related factors after mild brain injury: mild, decreasing fatigue with no other problems (class 1), high persistent fatigue with cognitive problems (class 2) and mood problems (class 3), and improving fatigue with mood problems (class 4). Class 1 comprised more than half of the participants and had the most favourable outcome overall, showing improvement in their fatigue with no problems on any of the other variables. Classes 2–4 all experienced clinically relevant fatigue (which did not improve for classes 2 and 3) but with different combinations of other symptoms, either fatigue with only cognitive problems (class 2), only mood problems (class 4) or both (class 3). Classes that experienced cognitive problems (2 and 4) also showed lower satisfaction in their societal participation.

The present study found a number of similarities to Rakers and colleagues’ trajectories (5). First, the proportion of participants with a good outcome in fatigue recovery in both studies was just over half. While these participants were spread across the first 2 classes in Rakers’ paper, the overall proportion of participants with mild fatigue was similar. Second, both the present study and Rakers et al. found that while 1 class experienced higher fatigue in combination with anxiety and depression, another class experienced high fatigue without any mood problems. This shows that while fatigue can correlate with mood problems, there are specific subgroups of patients who will experience fatigue without mood issues.

In contrast to Rakers et al. (5), this study also included cognitive complaints and societal participation in the MLCGA model. Interestingly, those who experienced fatigue in combination with cognitive problems were more likely to experience problems with societal participation than those who experienced only fatigue and mood problems. This could suggest that access to and enjoyment of social activities is more impeded by cognitive problems than the other measured symptoms. This would be in line with previous research into impairments in leisure activities after ABI, which found that subjective cognitive complaints (specifically reduced speed of mental processing and executive impairments) had the largest effect on the ability for participants to engage in preferred leisure activities (20).

Of the 4 classes, only class 4 (improving fatigue with mood problems) showed an increase in fatigue between 6 and 12 months. In previous studies the rise in fatigue at this time point has been attributed to changes in social life (e.g. return to work) (4), which could be driving the change here too. For class 1 (decreasing fatigue), their overall recovery by this time point could protect them from a fatigue increase regardless of social changes. However, for classes 2 and 3 (persistent fatigue with other problems), given that their fatigue levels were already high at 6 months, the severity scoring may not be sensitive enough to capture any changes to social environment or, alternatively, they may have been less likely to return to work at this time given their persistent fatigue. Finally, class 4, while showing improvements in fatigue up to 6 months, still had mood problems throughout, meaning that they could have been more susceptible to an increase in fatigue in response to social changes.

The present study also showed that certain demographic and injury characteristics predicted membership of specific classes. There was a higher chance of being in class 2 (high persistent fatigue with primarily cognitive problems) if a participant was female. This class experienced problems with fatigue, cognitive complaints and societal participation, but not anxiety or depression. While this aligns with research indicating that fatigue after brain injury is more prevalent among women (21), it is interesting that this group did not experience mood problems, as previous research suggests that women tend to have higher rates of anxiety and depression after brain injury (22).It could be, however, that while classes 3 (high persistent fatigue with both mood and cognitive problems) and 4 (initially improving moderate fatigue and primarily mood problems) in the present research were the classes that had the highest rates of anxiety and depression, they also represent a much smaller number of patients, reducing the likelihood of observing differences in sex in these classes.

There was also a higher chance of older patients being in class 2 than class 1 (mild, decreasing fatigue with no other problems) or 3. Class 2 experienced problems with societal participation and cognition, which are both associated with normal ageing. This finding is particularly in line with research showing that older age correlates with problems after ABI in both cognition (23, 24) and societal participation (25, 26).

Rakers et al. (5) found that low education level predicted membership in a class with an unfavourable recovery outcome. While this was not seen in the current study, potentially due to the imbalance in group sizes, given the previous findings of Rakers et al., consideration could be given to the accessibility of treatments for fatigue and other symptoms for patients with a lower education level, given their potential for worse recovery outcomes.

While it was found that those with mTBI were more likely to be in class 1 than class 4, it is important to note that this finding is likely influenced by the group sizes. Class 1 represented 53% of the population and mTBI patients formed 78% of the current cohort. Therefore, this finding could be being driven by a smaller number of stroke patients overall than a difference in recovery outcome based on injury type. As injury type did not predict membership differently between classes 1 and 2, there do not seem to be differences in fatigue severity based on injury type. Consequently, early fatigue screenings could benefit both mild stroke and mTBI patients in identifying a need for treatment.

### Strengths and limitations

This study adds to the existing evidence relating to recovery after brain injury. By identifying latent classes of recovery on multiple outcomes this research provides a richer picture of what recovery from brain injury can look like for different individuals. In particular, one strength of this study is the inclusion of cognitive complaints and social participation as these variables have not been included before in latent class analysis on fatigue recovery after brain injury, allowing for a novel view of the relationships between these variables. A further strength is the novel inclusion of both mild stroke and mTBI patients in the model. Both groups often use the same resources for treatment and while past research has shown that mood and cognitive outcomes are comparable between groups (8), the current study provides further support for this finding with regard to fatigue.

Conversely, there are a number of limitations that should be considered with the current research. First, the present analysis does not provide insight into the causal relationships between variables. Identifying directionality here could help to inform whether treatments for some symptoms should be started earlier than others. Furthermore, while the current study included cognitive and mood-based variables in the MLCGA model, other somatic illnesses and psychological variables that can influence fatigue were not included. As Rakers et al. (5) found that coping style was connected to fatigue, future research should also consider psychological variables when assessing recovery from ABI.

Finally, fatigue was measured on a severity scale and, thus, the separate dimensions of fatigue cannot be separated in the current model. Fatigue is a multi-dimensional variable with different aspects such as physical, mental and emotional fatigue (27). It could be that the difference seen in the combinations between fatigue and other symptoms is reflective of the different types of fatigue, e.g. cognitive fatigue could correlate with cognitive complaints, whereas emotional fatigue could be more closely related to anxiety and depression. While there is limited research into the correlates of specific fatigue domains after ABI, there is sufficient evidence to show that the different domains of fatigue can be experienced separately and therefore should be considered in future research (28). As the present study measured only overall fatigue severity it is not possible to disentangle how the domains of fatigue may have affected the comorbidity with other symptoms. Future research should investigate the comorbidity of the different types of fatigue with other post-injury symptoms.

### Clinical implications

Given that fatigue was mostly seen in connection with other symptoms it is important to consider treatment of a more integrated nature, approaching multiple symptoms simultaneously, depending on the individual’s profile. Targeting fatigue by itself without considering other symptoms could lead to a neglect of other affected domains and result in an incomplete recovery. Recent best practice guidelines for care after stroke and mTBI have highlighted mood problems, cognitive complaints and fatigue as three areas in particular that contribute to worse outcomes in recovery (29, 30). In their recommendations, early screening is highlighted as being particularly important for improving longer term outcomes. The results of the current study support these recommendations. Given that the classes that showed less overall recovery (classes 2, 3 and 4) were already showing heightened levels of their respective problems at the first time point, early screenings should happen as soon as possible, but at least within 6 weeks of ABI.

Following screening, early treatment would be recommended. Although it is not known in the present research what treatments each class was receiving, nor the timing of treatment, there is some evidence from previous research to suggest that early intervention after brain injury is beneficial for long-term rehabilitation outcomes (31, 32).

Furthermore, in cases where individuals show fatigue in combination with cognitive complaints, it would be important to look at whether any complaints directly impact societal participation. As previous research has shown that opportunities to participate in social events can be a good motivator for overcoming fatigue (7), treatment that specifically addresses cognitive complaints might also ease restrictions on social activities and could reduce the isolating side effect of fatigue. Future research should also investigate the causal directions of the post-injury symptoms to highlight symptoms that could be targeted early to reduce or even prevent other symptoms.

Finally, follow-ups between 6 and 12 months could help to highlight where symptoms might start to return. In the present study, class 4 experienced a rise in fatigue between 6 and 12 months back to clinically significant levels. Following up with patients during this period could help to reduce or prevent a secondary spike in fatigue and other symptoms.

### Conclusion

The current research provides further evidence of the variation in recovery trajectories for fatigue following mild ABI. Half the participants had a favourable outcome, showing a decrease in fatigue over time with no other problems, but the remaining classes were divided between those who had fatigue with only mood problems, only cognitive problems or both in the presence of lower participation satisfaction. While the 4 trajectories add further evidence of the high comorbidity of fatigue, mood problems and cognitive complaints, it is clear that the specific combinations differ between individuals. Given this variability, treatment after ABI should be based on individualised post-injury symptom profiles.

## Supplementary Material

TRAJECTORIES OF FATIGUE AND RELATED OUTCOMES FOLLOWING MILD ACQUIRED BRAIN INJURY: A MULTIVARIATE LATENT CLASS GROWTH ANALYSIS

TRAJECTORIES OF FATIGUE AND RELATED OUTCOMES FOLLOWING MILD ACQUIRED BRAIN INJURY: A MULTIVARIATE LATENT CLASS GROWTH ANALYSIS
