# Supplementary material for: TRAJECTORIES OF FATIGUE AND RELATED OUTCOMES FOLLOWING MILD ACQUIRED BRAIN INJURY: A MULTIVARIATE LATENT CLASS GROWTH ANALYSIS
Source: J Rehabil Med. 2024 Mar 20;56:32394. doi: 10.2340/jrm.v56.32394 (PMC10985494; doi:10.2340/jrm.v56.32394)

Supplementary material has been published as submitted. It has not been copyedited, typeset or checked for scientific content by Journal of Rehabilitation Medicine

Fig. SI. Distribution of fatigue severity over time

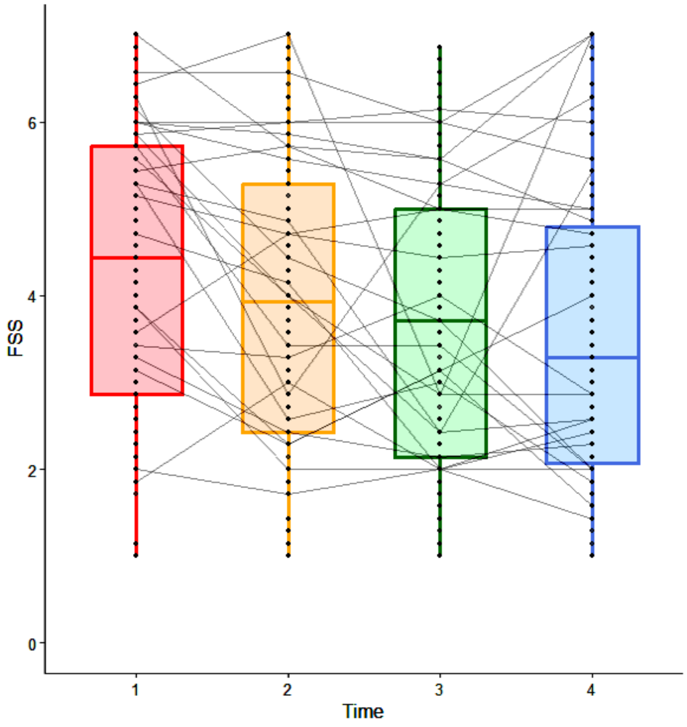

FSS-7 scores over the four time points. The central line in the box represents the median with the outer edges of the box showing the 25th and 75th percentiles. Each dot represents a participant and the change in individual fatigue scores are shown as black lines for a subset of participants (first 25).

Fig. S2. Probability of class membership based on demographic characteristics

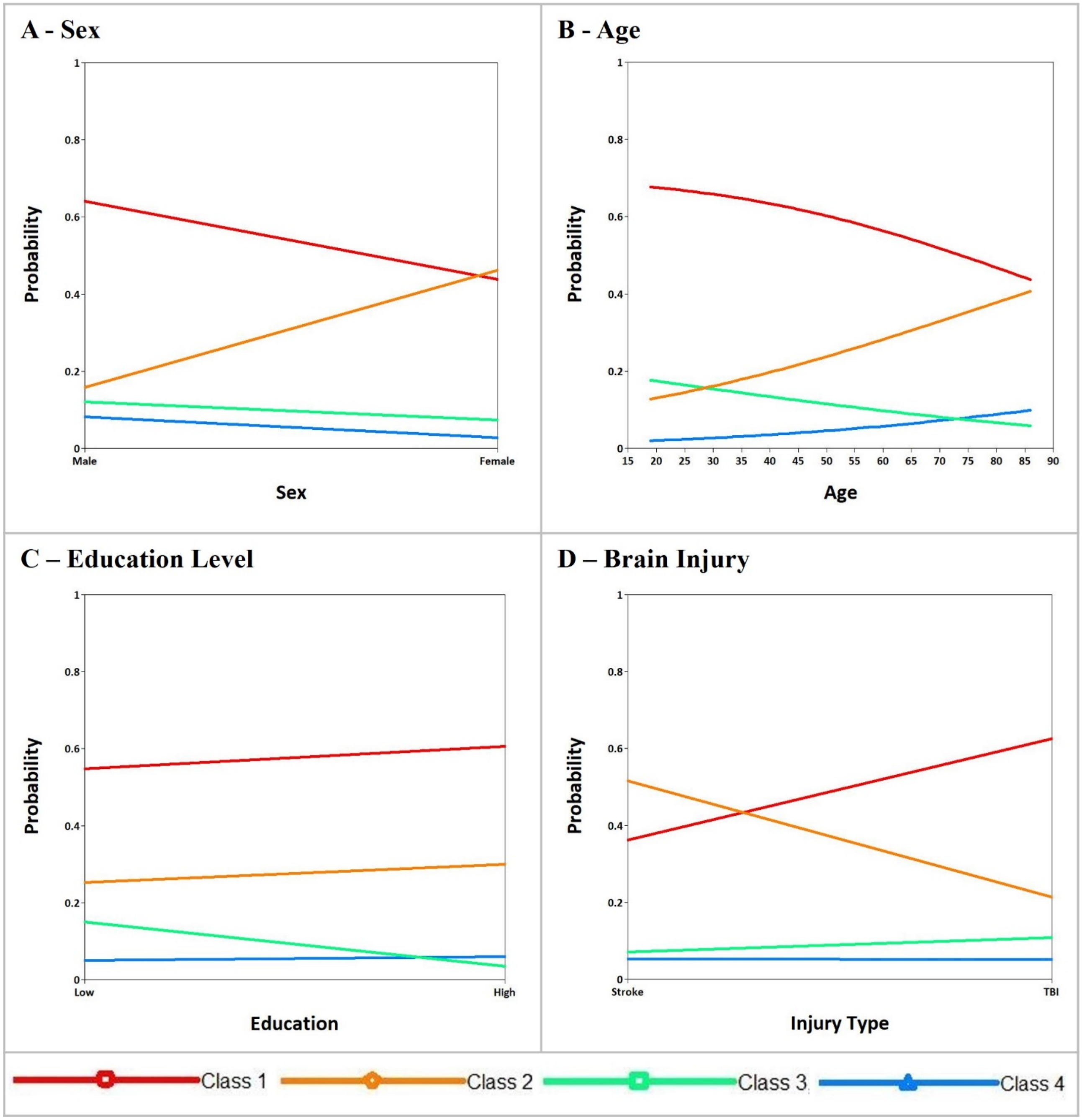

Supplement: TRAJECTORIES OF FATIGUE AND RELATED OUTCOMES FOLLOWING MILD ACQUIRED BRAIN INJURY: A MULTIVARIATE LATENT CLASS GROWTH ANALYSIS [file JRM-56-32394-s1.pdf]
