# Supplementary material for: TRAJECTORIES OF FATIGUE AND RELATED OUTCOMES FOLLOWING MILD ACQUIRED BRAIN INJURY: A MULTIVARIATE LATENT CLASS GROWTH ANALYSIS
Source: J Rehabil Med. 2024 Mar 20;56:32394. doi: 10.2340/jrm.v56.32394 (PMC10985494; doi:10.2340/jrm.v56.32394)
Supplement: TRAJECTORIES OF FATIGUE AND RELATED OUTCOMES FOLLOWING MILD ACQUIRED BRAIN INJURY: A MULTIVARIATE LATENT CLASS GROWTH ANALYSIS [file JRM-56-32394-s2.pdf]

Supplementary material has been published as submitted. It has not been copyedited, typeset or checked for scientific content by Journal of Rehabilitation Medicine

Table SI. Count and percentage of missing data for each variable per time point

**Variable**	**Missing**	
	N	%
FSS T0	3	1.5
FSS T1	16	8.5
FSS T2	23	12.7
FSS T3	33	19.3
HADS_A T0	6	3.0
HADS_A T1	23	12.7
HADS_A T2	30	17.2
HADS_A T3	43	26.7
HADS_D T0	8	4.1
HADS_D T1	24	13.3
HADS_D T2	31	17.9
HADS_D T3	44	27.5
Cognition T0	7	3.6
Cognition T1	20	10.9
Cognition T2	27	15.3
Cognition T3	33	19.3
USER-P Res T0	7	3.6
USER-P Res T1	26	14.6
USER-P Res T2	31	17.9
USER-P Res T3	39	23.6
USER-P Sat T0	5	2.5
USER-P Sat T1	18	9.7
USER-P Sat T2	23	12.7
USER-P Sat T3	37	22.2

FSS: Fatigue Severity Scale; HADS_A: Hospital Anxiety Depression Scale – Anxiety Subscale; HADS_D: Hospital Anxiety Depression Scale – Depression Subscale; USER-P Res: Utrecht Scale for Evaluation and Rehabilitation-Participation – Restrictions Subscale; USER-P Sat: Utrecht Scale for Evaluation and Rehabilitation-Participation – Satisfaction Subscale.

Table SII. Mean fatigue scores on the FSS-7 and FSS-9 for each time point

**Time**	**FSS-7**<br>Mean ± SD	**FSS-9**<br>Mean ± SD
0	4.32 ± 1.63 (N=201)	4.41 ± 1.45 (N=200)
1	3.84 ± 1.73 (N=188)	3.97 ± 1.58 (N=188)
2	3.68 ± 1.71 (N=181)	3.89 ± 1.51 (N=180)
3	3.54 ± 1.71 (N=171)	3.74 ± 1.58 (N=171)

Table SIII. P-values for the slope and quadratic effects of each class curve per variable

**Variable**	**Class**							
	1		2		3		4	
	Linear	Quadratic	Linear	Quadratic	Linear	Quadratic	Linear	Quadratic
Fatigue	.000***	.000***	.329	.809	.434	.542	.033*	.041*
Anxiety	.000***	.001**	.485	.73	.178	.078	.595	.558
Depression	.000***	.000***	.016*	.027*	.221	.564	.215	.397
Cognitive Complaints	.016*	.037*	.238	.150	.554	.414	.554	.862
Restrictions	.000***	.000***	.000***	.001**	.012*	.061	.544	.814
Satisfaction	.000***	.007**	.053	.076*	.754	.627	.783	.89

* p<.05; ** p<.01; *** p<.001
